# Single-cell RNA sequencing analysis revealed a potential association between ELK3 expression and the progression of multiple myeloma

**DOI:** 10.3389/fimmu.2026.1793391

**Published:** 2026-04-20

**Authors:** Chengcheng Song, Taowu Chen, Sijia Yu, Tianjiao Huang, Tianxin Chen, Cuicui Wang, Yanchen Liang

**Affiliations:** 1 School of Acupuncture and Tuina, Shandong University of Traditional Chinese Medicine, Jinan, China; 2 School of Stomatology, Southwest Medical University, Luzhou, China; 3 Clinical Medical College, Southwest Medical University, Luzhou, China; 4 The First School of Clinical Medicine, Heilongjiang University of Chinese Medicine, Harbin, China; 5 Wangjing Hospital of China Academy of Chinese Medical Sciences, Beijing, China; 6 Department of Hematology, The First Affiliated Hospital of USTC (West District), Anhui Provincial Cancer Hospital, University of Science and Technology of China, Hefei, China; 7 Department of Orthopedics, Affiliated Hospital of Shandong University of Traditional Chinese Medicine, Jinan, China

**Keywords:** monoclonal gammopathy of undetermined significance, multiple myeloma, scRNA-seq, smoldering multiple myeloma, tumor heterogeneity

## Abstract

**Background:**

Multiple myeloma (MM) typically evolves from monoclonal gammopathy of undetermined significance (MGUS) and smoldering multiple myeloma (SMM). Its progression is accompanied by significant tumor heterogeneity and immune microenvironment remodeling, and MM remains largely incurable despite therapeutic advances. Elucidating cellular heterogeneity and regulatory mechanisms involved in disease progression is critical for understanding MM pathogenesis.

**Methods:**

Single-cell RNA sequencing (scRNA-seq) provides important support for systematically elucidating the molecular mechanisms and immune regulatory pathways associated with the progression of MM. The scRNA-seq datasets from healthy donors (HD), MGUS, SMM, and MM patients were obtained from the GEO database to analyze plasma cell transcriptional heterogeneity within the bone marrow microenvironment. Plasma cell differentiation trajectories were inferred using pseudotime analysis, and intercellular communication and transcription factor regulatory networks were predicted using CellChat and SCENIC analyses. In addition, functional validation of *ELK3* was performed *in vitro* using MM cell lines RPMI 8226 and U266.

**Results:**

In this work, we used scRNA-seq to thoroughly evaluate the heterogeneity of MM. We identified a tumor-associated plasma cell subtype, C4 *TTN+* plasma cell subtype, which was predominant in SMM and MM and was significantly enriched throughout the intermediate and final phases of differentiation. We hypothesized that C4 *TTN+* plasma cell subtype may be associated with the progression of MM. We also found that the TGFb signaling pathway played a key role in the interaction between C4 *TTN+* plasma cell subtype and various cell types within the TME. Furthermore, *in vitro* experiments validated the positive regulatory effects of ELK3 on plasma cell proliferation, migration, and cell viability in MM.

**Conclusion:**

In summary, this study, based on scRNA-seq analysis, identified a C4 *TTN+* plasma cell subtype potentially associated with *ELK3*. This subtype may be associated with progression and immune evasion in MM, thereby providing a potential new direction for targeted immunotherapeutic strategies in MM.

## 1 Introduction

Multiple myeloma (MM) is a malignant plasma cell neoplasm characterized by the clonal expansion of aberrant plasma cells (1). Since 1990, the global incidence of MM has shown a sustained increase, with a particularly marked rise among the elderly population (2, 3), thereby imposing a substantial burden on public health systems (4). MM typically evolves through two premalignant stages, namely monoclonal gammopathy of undetermined significance (MGUS) (5) and smoldering multiple myeloma (SMM) (6), both of which generally lack overt clinical manifestations (7). Epidemiological studies indicate that approximately 5% of adults are diagnosed with MGUS (8), with an estimated annual risk of progression to MM of about 1% (9). Furthermore, nearly one-third of patients with SMM ultimately progress to MM, with an annual progression risk of approximately 10% (10, 11). MM is frequently associated with a broad spectrum of clinical complications, including hypercalcemia, renal dysfunction, anemia, bone pain, and profound immunosuppression (12, 13). Accumulating evidence indicates that immune dysregulation plays a pivotal role in the initiation and progression of MM (14). During the MGUS or SMM stages, patients are generally maintained in a state of relative immune homeostasis. However with the progressive accumulation of genetic alterations, functional exhaustion of T cells, and dynamic remodeling of the tumor microenvironment (TME), this immune equilibrium is gradually disrupted. The resulting immunosuppressive TME facilitates immune evasion and ultimately drives the progression of MM (15).

Autologous stem cell transplantation remains one of the standard therapeutic strategies for eligible patients with MM. This therapy not only effectively reduces tumor burden but also partially restores antitumor immune function, thereby significantly prolonging patient survival (16, 17). With the rapid progress of targeted immunotherapy in the treatment of MM, new treatment strategies, including monoclonal antibodies, bispecific antibodies, and immune checkpoint inhibitors, are constantly emerging, providing more options for patients with relapsed or refractory MM (18). Nevertheless, MM remains largely incurable in clinical practice and is characterized by a high rate of relapse (19, 20). Consequently, elucidating the key molecular mechanisms underlying MM progression, delineating disease heterogeneity, and identifying critical drivers of disease advancement are of substantial scientific importance and clinical relevance for early risk stratification of high-risk patients, implementation of precision therapeutic interventions, and improvement of overall patient outcomes.

Single-cell RNA sequencing (scRNA-seq) technology can characterize the transcriptome features of cells at relatively high resolution, thereby revealing cellular heterogeneity and identifying cell subtypes with potential biological significance in complex tissues (21). This technology has been widely used in research on various cancers, providing strong support for a deeper understanding of the dynamic changes in the occurrence and development of cancer. In this study, we used scRNA-seq technology to systematically analyze healthy donors (HD), MGUS, SMM, and MM samples, aiming to reveal cellular heterogeneity and potential molecular mechanisms in the progression of MM, and to identify key malignant subpopulations and regulatory factors. This study aims to provide potential intervention targets for further delaying the malignant progression of MM and improving patient prognosis.

## 2 Materials and methods

### 2.1 Source of data and preprocessing

We obtained 14 samples from patients with MM and its precancerous lesions (MGUS and SMM) and 5 bone marrow aspiration samples from HD from the GEO database (https://www.ncbi.nlm.nih.gov/geo/) (GSE271107) (22). Raw expression matrices were processed using the Seurat R package (v4.3.0) (23). Potential doublets were subsequently removed using the DoubletFinder package (v2.0.3) (24, 25). Quality control filtering was applied to remove low-quality cells. Cells were retained if they met the following conditions: mitochondrial gene proportion < 25%; 300 < nFeature < 6,000; 500 < nCount < 100,000; and hemoglobin gene proportion < 5% (26, 27).

Data normalization was performed using the “NormalizeData”. The “FindVariableFeatures” and “ScaleData” function were used to identify the top 2000 highly variable genes and perform standardization (28). The batch effect was reduced by using the Harmony package (v0.1.1), and the top 30 principal components were chosen for further analysis (29, 30).

### 2.2 Cell type identification

After dimensionality reduction and clustering, the cell populations were shown using the uniform manifold approximation and projection (UMAP) (31, 32). The CellMarker database (http://xteam.xbio.top/CellMarker/) (33) was then used to annotate cell clusters. To further elucidate the dynamic changes of plasma cells during MM progression, plasma cells were subjected to an additional round of dimensionality reduction, clustering, and subtype annotation (34).

### 2.3 Differentially expressed genes enrichment and AUCell analysis

The “FindAllMarkers” function was applied to identity differentially expressed genes (DEGs) across cell clusters (35). The clusterProfiler (v4.6.2) and SCP (v0.4.8) packages were then used to conduct functional enrichment analysis of the DEGs in order to investigate putative biological processes. Gene Ontology (GO) and Kyoto Encyclopedia of Genes and Genomes (KEGG) enrichment analyses (36) were employed to characterize the enrichment of specific functional terms. Gene Set Enrichment Analysis (GSEA) (37, 38) was further performed to explore gene regulatory relationships (39). Finally, the scgmt package (v0.0.3) was used to construct AUCell matrices, thereby quantifying the activity and enrichment of particular groups of genes in individual cells.

### 2.4 Analysis of plasma cell differentiation trajectories and stemness-related characteristics

CytoTRACE2 (v1.1.0) was applied to score cellular stemness and assess the differentiation potential of plasma cell subtypes. Subsequently, a pseudotime model was constructed using the Monocle package (v2.24.0) to delineate cellular differentiation trajectories. Furthermore, the Slingshot package (v2.6.0) was employed to infer lineage trajectories among plasma cell subtypes, thereby providing a comprehensive characterization of their differentiation relationships.

### 2.5 Cell communication analysis

The CellChat software (v1.6.1) was utilized to create intercellular communication networks and to conduct ligand-receptor interaction research to clarify the relationships between various cell types (40–42). In addition, “netVisual_diffInteraction” function (43) was used to compare the strength of interactions between different cells, enabling the identification of significant signaling pathways that might be connected to MM progression.

### 2.6 Construction of gene regulatory networks

To characterize transcription factors (TF) regulatory patterns in plasma cells, we employed the SCENIC package (v1.3.1) to construct gene regulatory networks, deduce co-expression modules among TFs and their target genes, and identify key regulons (44). The “AUCell” function was then used to calculate regulator activity at the single-cell level. Based on regulon-specific scores and AUCell values, the top five TFs were selected for visualization (45).

### 2.7 Cell culture

The MM cell lines RPMI 8226 (Cat. No. TCHu158) and U266 (Cat. No. TCHu189) used in this study were obtained from the Cell Bank of the Chinese Academy of Sciences (Shanghai, China). Cells were cultured in RPMI-1640 medium supplemented with 10% fetal bovine serum (FBS) and 1% penicillin-streptomycin solution, and kept in a humidified incubator at 37 °C with 5% CO_2_ (46). Cells were passaged every 2–3 days to maintain logarithmic growth (47).

### 2.8 CRISPR/Cas9-mediated *ELK3* silencing

CRISPR/Cas9-mediated gene silencing was performed using a lentiviral system to investigate the function of *ELK3* in MM plasma cells. Two distinct single-guide RNAs (sgRNAs) targeting human *ELK3* were designed and individually cloned into the lentiCRISPRv2 vector in accordance with the conventional cloning procedure.

The constructed lentiCRISPRv2-*sgELK3* plasmid and packaging plasmids psPAX2 and pMD2.G were co-transfected into HEK293T cells. Viral supernatants were collected and filtered at 48 and 72 hours after transfection and used to infect RPMI 8226 and U266 cells. To create stable *ELK3*-knockdown cell lines, cells were selected after 48 hours of infection and treated with 1 μg/mL puromycin for 5–7 days. Prior to subsequent functional tests, knockdown efficiency was confirmed at the mRNA level using quantitative real-time PCR.

### 2.9 Quantitative real-time PCR

To verify the effectiveness of *ELK3* gene knockdown in MM cell lines, total RNA was extracted using TRIzol Reagent (48). After determining the concentration and purity of the RNA, reverse transcription was performed to synthesize complementary DNA (cDNA). Quantitative real-time PCR was then performed.

### 2.10 Cell proliferation assay (CCK-8)

Cell viability following *ELK3* knockdown was assessed using the Cell Counting Kit-8 (CCK-8) according to the manufacturer’s instructions. Briefly, RPMI 8226 and U266 cells were seeded into sterile 96-well flat-bottom plates. Three experimental groups were established: an sgRNA control group (sg-Ctrl), and two *ELK3*-targeting groups transduced with independent sgRNAs (sg-ELK3#1 and sg-ELK3#2). Each group was set up in six technical replicates. Cells were incubated at 37 °C in a humidified incubator containing 5% CO_2_. At the indicated time points, 10 μL of CCK-8 solution was directly added to each well, and the mixture underwent incubation at 37 °C for two hours. A SpectraMax i3x microplate reader was used to measure the absorbance at 450 nm. Cell viability was expressed as the mean optical density at 450 nm (OD_450_) of triplicate wells.

### 2.11 Colony formation assay

Long-term proliferative capacity following *ELK3* knockdown was evaluated using a colony formation assay. Briefly, RPMI 8226 and U266 cells from the sg-Ctrl, sg-ELK3#1, and sg-ELK3#2 groups were seeded in 6-well plates, and 2 mL of RPMI-1640 medium containing 10% FBS was added to each well. Cells were gently resuspended to ensure uniform distribution and incubated at 37 °C with 5% CO_2_. The culture medium was changed every 3–4 days to ensure ideal nutritional conditions. After the colonies were fixed at room temperature for 15 minutes, they were rinsed twice with PBS and then stained with crystal violet solution for 20 minutes. After staining, gently wash away excess dye with distilled water and air dry at room temperature.

### 2.12 Cell migration assay (Transwell)

Cell motility was evaluated using Transwell^®^ polycarbonate membrane inserts. To minimize proliferation-related confounding, cells were serum-starved for 6 hours prior to seeding.

RPMI 8226 and U266 cells from the sg-Ctrl, sg-ELK3#1, and sg-ELK3#2 groups were harvested in the logarithmic phase, viability confirmed to be > 90% by trypan blue exclusion, and counted with a hemocytometer. For each insert, 2 × 10^5^ cells were resuspended in 200 μL of serum-free RPMI-1640 and introduced into the upper chamber; the bottom chamber was filled with 600 μL of RPMI-1640 supplemented with 10% FBS as the chemoattractant. During a 24-hour incubation period at 37 °C and 5% CO_2_, non-migrated cells on the upper surface were carefully removed with a sterile cotton swab. Membranes were rinsed in PBS, fixed in 100% methanol for 10 min at room temperature, washed, and stained with 0.1% crystal violet for 20 minutes. After using distilled water to remove any remaining dye, the inserts were allowed to air dry.

### 2.13 Apoptosis analysis

RPMI-8226 and U266 cells from the sg-Ctrl, sg-ELK3#1, and sg-ELK3#2 groups were harvested during the logarithmic growth phase. A non-transduced negative control group (NC) was included to evaluate potential effects of the transduction procedure or vector system on basal cell viability. Cells were subsequently washed twice with ice-cold phosphate-buffered saline and resuspended in binding buffer. A 100 μL of cell suspension was aspirated from each sample and placed in a test tube. The cells were stained with the Annexin V–FITC/PI apoptosis kit at room temperature in the dark for 15 minutes. Binding buffer was then added to each tube, and apoptosis detection and analysis were immediately performed using a BD FACSCanto II flow cytometer. The FlowJo software (v10.8.1) was used to process the data. For statistical study, the proportion of all apoptotic cells (early + late apoptosis) was utilized.

### 2.14 Statistical analysis

R software (v4.3.2) and GraphPad Prism software (v9.0) were used for all statistical analyses. Unless otherwise indicated, data are presented as mean ± standard deviation (SD) (49). All experiments were independently repeated at least three times (50).

All quantitative data were visualized using GraphPad Prism, and p-values were denoted as follows: p < 0.05 (*), p < 0.01 (**), and p < 0.001 (***) (51). Statistical significance was set at p < 0.05 (52). Graphs and bar plots depict mean values with error bars representing SD unless otherwise specified.

## 3 Results

### 3.1 scRNA-seq analysis in the progression of MM

**Figure 1:**
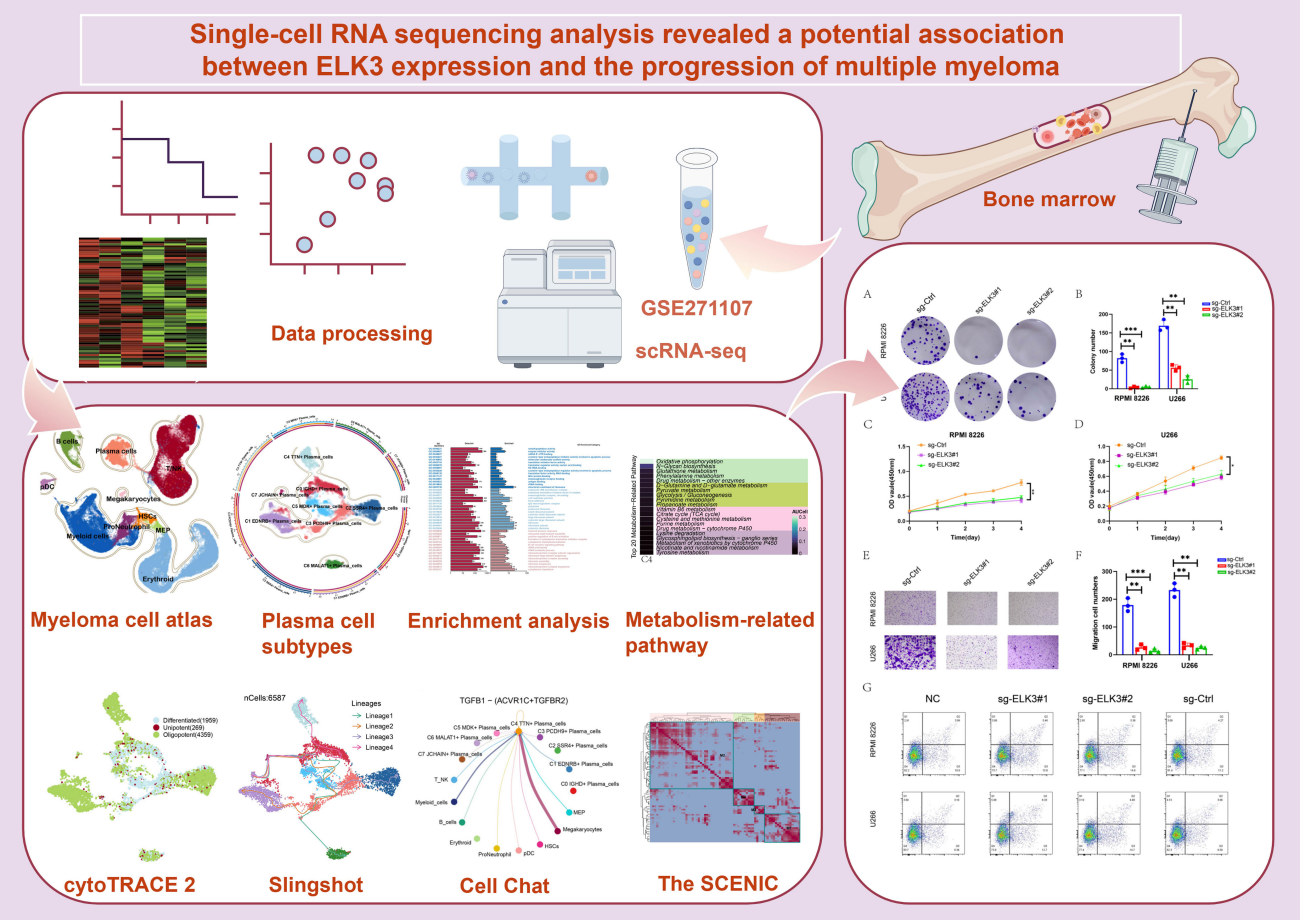
MM scRNA-seq: analysis workflow. The scRNA-seq analysis process for MGUS, SMM, MM, and HD included data processing, identification of key cell subtypes, pseudotime analysis, cell communication analysis, transcription regulatory network inference, and *in vitro* experimental validation.

The scRNA-seq analysis was performed on samples from six patients with MGUS, four patients with SMM, four patients with MM, and five HD. In addition, *in vitro* experiments were conducted to validate the functional role of *ELK3.* The overall study design and analytical workflow were illustrated in **Figure 1**.

**Figure 2:**
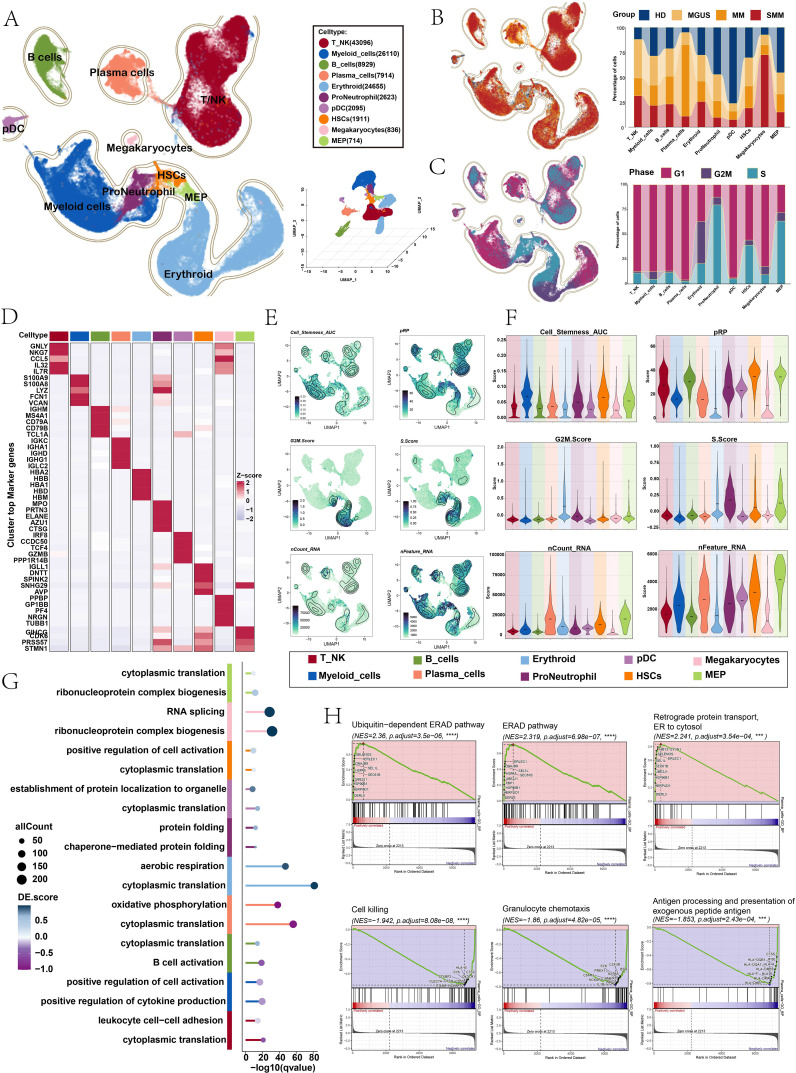
Identification of cell types in the samples using 10× scRNA-seq. **(A)** The UMAP plot visualized the single-cell lineages identified in this study. Distinct colors represented different cell types. The bottom-right panel illustrated the 3D spatial distribution of these cells. **(B, C)** The UMAP plots were colored by groups (top) and phases (bottom), and the bar charts showed the approximate proportions of different groups (top) and phases (bottom) in the ten cell types. **(D)** The heatmap depicted the expression patterns of the top five marker genes for each of the ten cell types. **(E, F)** The contour and violin plots illustrated the distribution and scores of cell stemness AUC, pRP, G2/M score, S score, nCount RNA, and nFeature RNA across the ten cell types. **(G)** The lollipop plot presented the GO enrichment analysis of key DEGs in the ten cell types. **(H)** GSEA (GO-BP) of plasma cells.

Following stringent quality control, a total of 118,883 cells were retained for subsequent analysis. Based on the expression of established marker genes, these cells were annotated into cell populations, including plasma cells, B cells, plasmacytoid dendritic cells (pDCs), T and NK cells (T/NK), megakaryocytes, myeloid cells, hematopoietic stem cells (HSCs), erythroid cells, pro-neutrophils, and megakaryocyte-erythroid progenitors (MEPs) (**Figure 2A**). Plasma cells were predominantly derived from MM samples, and the majority were in the G1 phase of the cell cycle (**Figures 2B, C**). For these ten cell types, the top five marker genes for each population were further identified (**Figure 2D**). These genes were consistent with previously reported cell type-specific markers. To further characterize the differences among these cell populations, we visualized the spatial distribution patterns of cell stemness AUC, pRP, G2/M phase, S phase, nCount RNA, and nFeature RNA, and compared AUCell scores across different cell types (**Figures 2E, F**). In addition, we performed GO-BP enrichment analysis. The results showed that oxidative phosphorylation and cytoplasmic translation were particularly prominent in plasma cells (**Figure 2G**). Next, GSEA revealed the upregulated and downregulated pathways in plasma cells. As shown in **Figure 2H**, plasma cells exhibited upregulation of pathways including retrograde protein transport from the endoplasmic reticulum (ER) to the cytosol, the ER-associated degradation (ERAD) pathway, and the ubiquitin-dependent ERAD pathway, whereas pathways related to cell killing, granulocyte chemotaxis, and antigen processing and presentation of exogenous peptide antigens were downregulated.

### 3.2 Heterogeneity of plasma cell subtypes

**Figure 3:**
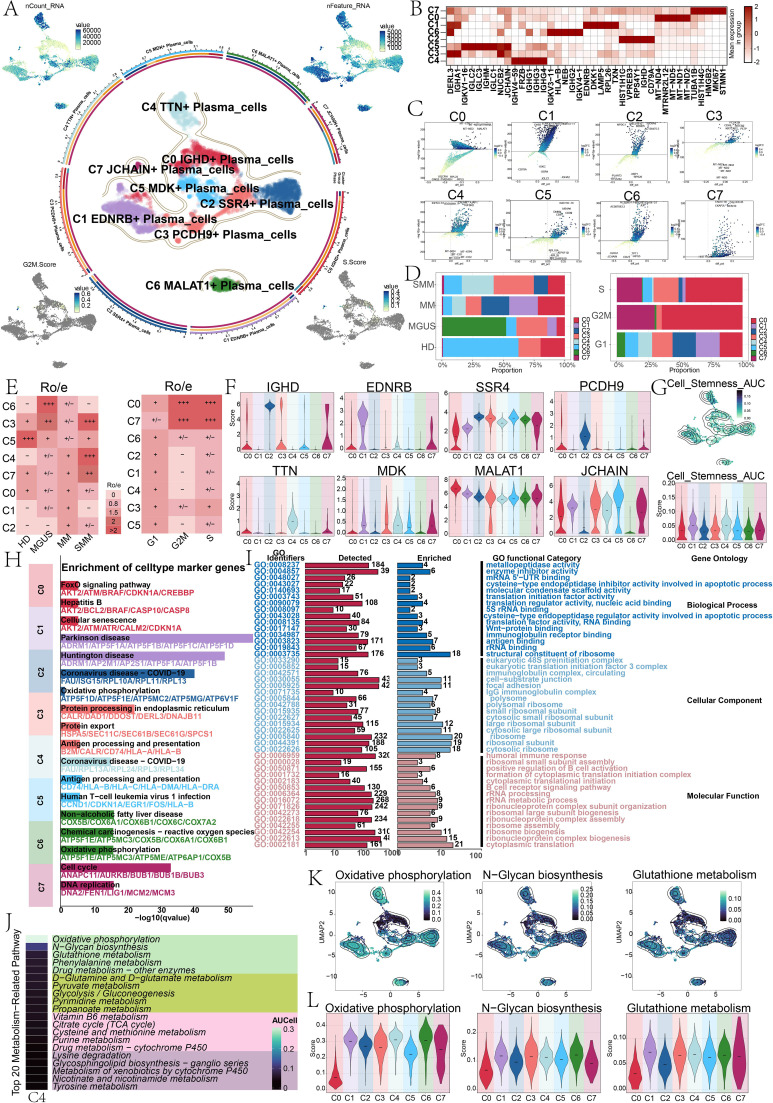
Single-cell landscape of plasma cell subtypes. **(A)** The circular plot illustrated the distribution of plasma cell subtypes, with the three concentric circles from outer to inner representing the approximate proportions of subtypes, groups, and phases. The UMAP plots at the four corners depicted the expression and distribution patterns of nCount RNA, nFeature RNA, G2M score, and S score. **(B)** The heatmap displayed the expression levels of marker genes across eight plasma cell subtypes, with deeper colors indicating higher expression. **(C)** The volcano plot demonstrated the differential gene expression profiles among the eight subtypes. **(D)** The bar plots presented the approximate percentages of the eight subtypes across different groups (left) and phases (right). **(E)** The heatmap illustrated the preference of the eight plasma cell subtypes for distinct groups (left) and phases (right), where Ro/e denoted the ratio of observed to expected cell counts. **(F)** The violin plots depicted the AUCell scores of named genes (*IGHD, EDNRB, SSR4, PCDH9, TTN, MDK, MALAT1, JCHAIN*) across the eight plasma cell subtypes. **(G)** The contour plot (top) and violin plot (bottom) demonstrated the distribution and scores of cell stemness AUC. **(H)** The bar plot showed the KEGG enrichment results for the eight plasma cell subtypes. **(I)** GO enrichment analysis results of C4 *TTN+* plasma cell subtype. **(J)** The heatmap displayed the top 20 metabolic pathways of C4 *TTN+* plasma cell subtype. **(K, L)** The contour plots and violin plots illustrated the distribution and AUCell scores of the top three metabolic pathways in C4 *TTN+* plasma cell subtype (oxidative phosphorylation, N-glycan biosynthesis, and glutathione metabolism).

To further clarify the crucial part that plasma cells play in the development of MM, we performed another cluster analysis on 6,587 plasma cells, and classified them into eight plasma cell subtypes. We then designated each subtype based on its significantly expressed genes: C0 *IGHD+* plasma cell subtype, C1 *EDNRB+* plasma cell subtype, C2 *SSR4+* plasma cell subtype, C3 *PCDH9+* plasma cell subtype, C4 *TTN+* plasma cell subtype, C5 *MDK+* plasma cell subtype, C6 *MALAT1+* plasma cell subtype, and C7 *JCHAIN+* plasma cell subtype (**Figure 3A**). Next, we further analyzed the top five marker genes of these eight plasma cell subtypes as shown in **Figure 3B**. The C4 *TTN+* plasma cell subtype mainly expressed *IGHV4-59, FRZB, IGHG1, IGHG3*, and *IGHG4*. To further characterize the differences between plasma cell subtypes, we showed differentially expressed genes that were upregulated and downregulated in eight plasma cell subtypes (**Figure 3C**) and compared the approximate percentages of the eight plasma cell subtypes across different groups and phases (**Figure 3D**).

We found that, compared to HD, C4 *TTN+* plasma cell subtype was a unique subtype in SMM and MM, and accounted for a relatively higher proportion in SMM and MM. Furthermore, the majority of C4 *TTN+* plasma cell subtype were in the G1 and S phases. The Ro/e heatmaps further revealed the groups and phases distribution preferences of the eight plasma cell subtypes, which were consistent with the above findings (**Figure 3E**). **Figure 3F** compared the AUCell scores of named genes within each plasma cell subtype, further characterizing the heterogeneity of plasma cell subtypes. To understand the plasma cell subtypes’ capacity for differentiation, we visualized the cell stemness AUC scores using contour plots and further compared them using violin plots. We found significant differences in stemness AUCell scores among the subtypes. The C1 *EDNRB+* plasma cell subtype and C4 *TTN+* plasma cell subtype exhibited relatively high stemness, followed by C0 *IGHD+* plasma cell subtype and C7 *JCHAIN+* plasma cell subtype, indicating that these subtypes possessed higher proliferation and differentiation potential (**Figure 3G**).

Enrichment analysis further revealed differences in biological functions among the subtypes. **Figure 3H** compared the differences in KEGG enrichment analysis among plasma cell subtypes, while **Figure 3I** showed the GO enrichment analysis results for C4 *TTN+* plasma cell subtype. We found that C4 *TTN+* plasma cell subtype was primarily concentrated in processes such as ribosomes, ribonucleoprotein synthesis, cytoplasmic translation, and ribosome structural components, which indicated that C4 *TTN+* plasma cell subtype had an exceptionally robust protein synthesis capacity. Cancer cell metabolism was closely related to cancer progression and outcome. **Figure 3J** illustrated the top 20 metabolic pathways associated with C4 *TTN+* plasma cell subtype, of which the top three were oxidative phosphorylation, N-glycan biosynthesis, and glutathione metabolism. To compare the differences in these three metabolic pathways among subtypes, we visualized them in contour plots and used violin plots for clearer comparison (**Figures 3K, L**).

### 3.3 Pseudotime trajectory and stemness analysis reveal the potential malignant progression of the C4 *TTN+* plasma cell subtype

**Figure 4:**
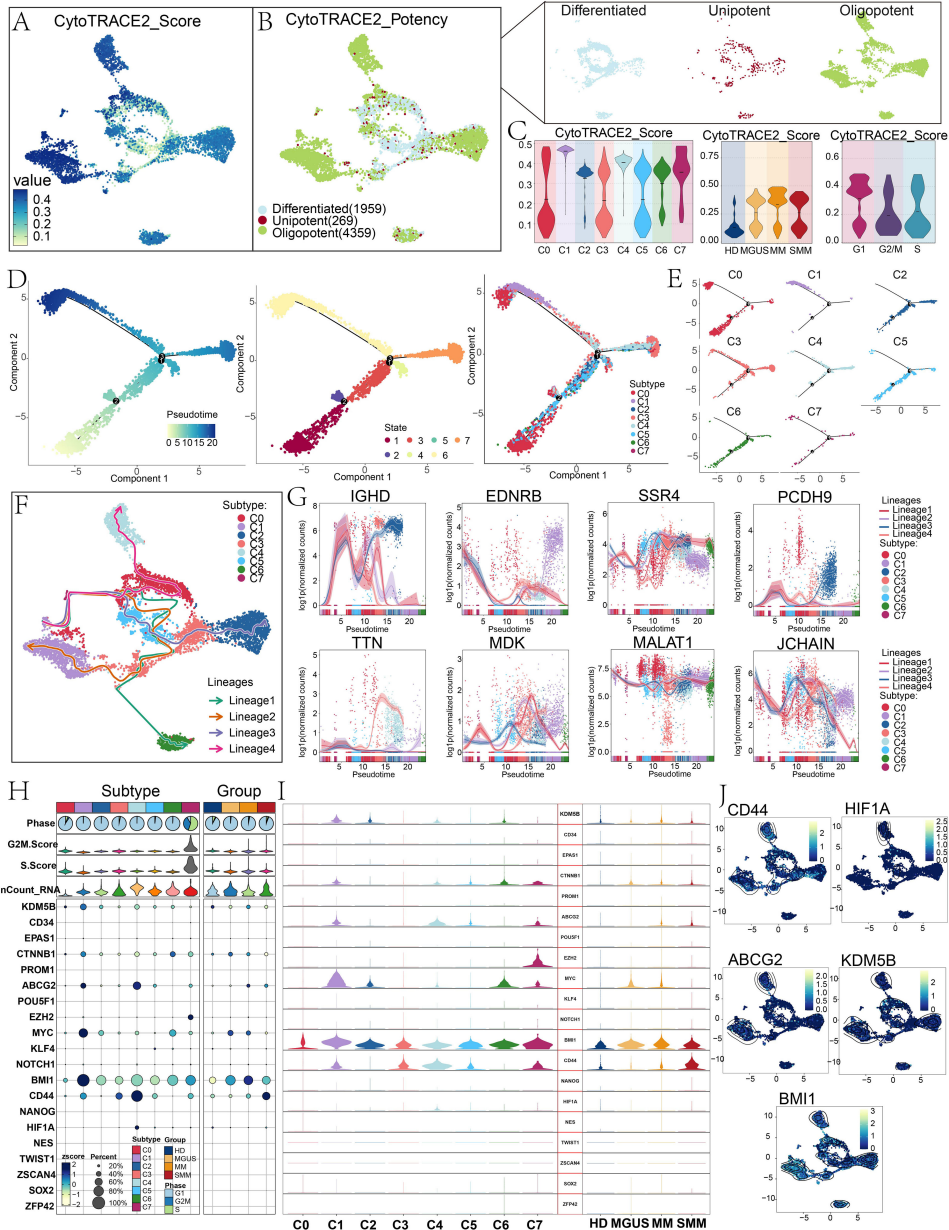
Trajectory analysis and stemness characteristics of plasma cell subtypes. **(A)** The UMAP plot reflected the differentiation potential scores of plasma cells. **(B)** The UMAP plot colored cells according to predicted differentiation potential categories, with the facet plots in the upper right further illustrating the distribution of differentiated, unipotent, and oligopotent states. **(C)** The violin plots compared the cytoTRACE2 scores across plasma cell subtypes (left), groups (middle), and phases (right). **(D, E)** Differentiation trajectories of plasma cell subtypes predicted by Monocle2, visualized by pseudotime (left), state (middle), and subtypes (right). The facet maps further displayed the trajectories colored by plasma cell subtypes. **(F)** The Slingshot analysis further inferred the differentiation trajectories of plasma cell subtypes: Lineage 1, Lineage 2, Lineage 3, and Lineage 4. **(G)** The dynamic trend scatter plots illustrated the changes of the named genes *IGHD, EDNRB, SSR4, PCDH9, TTN, MDK, MALAT1*, and *JCHAIN* along Lineage 1, Lineage 2, Lineage 3, and Lineage 4 with pseudotime. **(H)** The bubble plot depicted the expression of stemness-related genes in plasma cell subtypes and groups. Bubble colors were based on normalized data, and bubble size represented the percentage of cells expressing the gene. **(I)** The violin plot compared the expression levels of stemness-related genes across plasma cell subtypes and groups. **(J)** The contour plot displayed the expression levels of the top five marker genes in C4 *TTN+* plasma cell subtype.

Considering the differences in stemness among subtypes shown in **Figure 3G**, we further analyzed the stemness and differentiation potential of plasma cells and inferred their progression. We first used cytoTRACE2 to predict the differentiation potential of plasma cell subtypes. Notably, C1 *EDNRB+* plasma cell subtype and C4 *TTN+* plasma cell subtype had higher cytoTRACE2 scores compared to other subtypes, and most of them were oligopotent progenitors with high differentiation potential (**Figures 4A–C**). We then constructed pseudotime trajectories for the plasma cell subtypes using Monocle analysis. The findings demonstrated that C4 *TTN+* plasma cell subtype was primarily distributed in the middle and late stages of the pseudotime trajectory, with the majority belonging to states 6 and 7. Although the C1 *EDNRB+* plasma cell subtype was mostly found toward the pseudotime trajectory’s end, the C2 *SSR4+* plasma cell subtype, C5 *MDK+* plasma cell subtype, and C6 *MALAT1+* plasma cell subtype were primarily detected in the early and middle stages. Furthermore, C0 *IGHD+* plasma cell subtype exhibited a bimodal distribution, with some cells located at the beginning and others at the end of the pseudotime trajectory (**Figures 4D, E**).

Next, we used Slingshot analysis to more clearly visualize the four lineages of plasma cell subtypes: lineage 1 progressed sequentially through C1 *EDNRB+* plasma cell subtype, C7 *JCHAIN+* plasma cell subtype, C0 *IGHD+* plasma cell subtype, C5 *MDK+* plasma cell subtype, C3 *PCDH9+* plasma cell subtype, and C6 *MALAT1+* plasma cell subtype; lineage 2 followed the path of C1 *EDNRB+* plasma cell subtype, C7 *JCHAIN+* plasma cell subtype, C0 *IGHD+* plasma cell subtype, C5 *MDK+* plasma cell subtype, C3 *PCDH9+* plasma cell subtype, and C1 *EDNRB+* plasma cell subtype; lineage 3 followed the trajectory of C1 *EDNRB+* plasma cell subtype, C7 *JCHAIN+* plasma cell subtype, C0 *IGHD+* plasma cell subtype, C5 *MDK+* plasma cell subtype, C3 *PCDH9+* plasma cell subtype, and C2 *SSR4+* plasma cell subtype; lineage 4 began at C1 *EDNRB+* plasma cell subtype, progressed sequentially through C7 *JCHAIN+* plasma cell subtype and C0 *IGHD+* plasma cell subtype, and ultimately terminated at C4 *TTN+* plasma cell subtype (**Figure 4F**). Notably, the fluctuations in the expression of eight plasma cell subtype named genes along the pseudotime trajectory further support the above results (**Figure 4G**).

Cancer stem cells were capable of self-renewal and differentiation, and were significantly associated with tumor development, metastasis, and drug resistance (53, 54). We used bubble diagrams to display stemness-related genes in eight plasma cell subtypes and four sample types (**Figure 4H**), and violin diagrams to further compare changes in stemness gene expression (**Figure 4I**). The results showed that *BMI1* was expressed at certain levels across all subtypes and groups, *MYC* was most prominently expressed in C1 *EDNRB+* subtype and MGUS, and *HIF1A* was only expressed in C4 *TTN+* plasma cell subtype. *CD44, BMI1, ABCG2, HIF1A*, and *KDM5B* represented the five most highly expressed stemness-related genes in C4 *TTN+* plasma cell subtype. Their distribution was further illustrated in **Figure 4J**.

### 3.4 Cell communication network analysis predicted complex signal transduction among various cell types and identified key cancer-related signaling pathways

**Figure 5:**
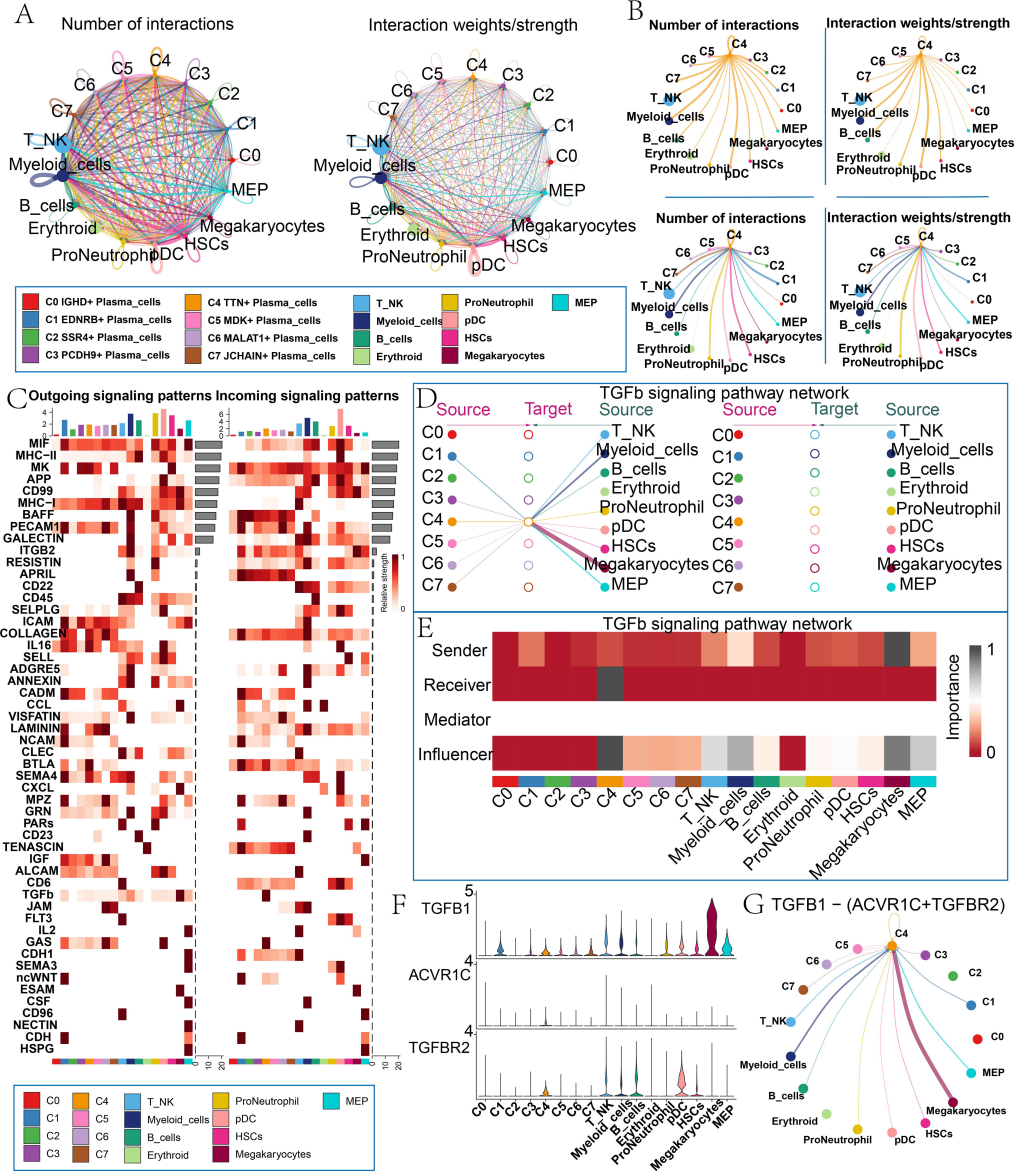
Analysis of communication between plasma cell subtypes and other cell types. **(A)** The circle plot showed the number (left) and strength (right) of interactions between different cell types. Thicker lines represented stronger interactions, and larger dots represented a greater number of cells. **(B)** The number (top left) and strength (top right) of interactions between C4 *TTN+* plasma cell subtype as a source cell and other cell types. The number (bottom left) and strength (bottom right) of interactions between C4 *TTN+* plasma cell subtype as a target cell and other cell types. **(C)** The heat map compared the outgoing and incoming signaling patterns between plasma cell subtypes and other cell types, as well as the relative strengths of the signaling pathways involved in each pattern. The vertical axis represented signaling pathway, and the horizontal axis represented cell type. Darker colors indicated stronger signal intensity. The bars above and to the right of the heat map represented the cumulative strength of the corresponding vertical and horizontal axes. **(D)** The hierarchical plot showed the communication patterns and signal strengths between plasma cell subtypes and between plasma cell subtypes and other cell types in the TGFb signaling pathway. Solid dots represented source cells, and hollow dots represented targets. Thicker lines indicated stronger communication. **(E)** The centrality score plot demonstrated the roles and expression levels of plasma cell subtypes and other cell types in the TGFb signaling pathway. **(F, G)** Violin plots and circle plots compared ligand and receptor interactions between plasma cell subtypes and other cell types in the TGFb signaling pathway.

To elucidate the communication patterns among different cell types and understand their regulatory role in disease progression, we performed CellChat analysis based on scRNA-seq data. We detected a complex and highly interactive communication network among the different cell types (**Figure 5A**), including plasma cell subtypes, T/NK cells, myeloid cells, B cells, erythroid cells, pro-neutrophils, pDCs, and HSCs. The C4 *TTN+* plasma cell subtype played a key role in signaling, acting as both a transmitter and a receiver, with particularly strong interactions with myeloid cells, megakaryocytes, HSCs, and B cells (**Figure 5B**). In addition, signaling pathways such as MK, IL16, BTLA and COLLAGEN had relatively high communication intensity in the outgoing signaling pattern of the C4 *TTN+* plasma cell subtype, while pathways such as TGFb, GAS, BAFF and APRIL were relatively prominent in the incoming signaling pattern (**Figure 5C**). Notably, we found that communication between C4 *TTN+* plasma cell subtype and other cell types was strongly enriched in the TGFb signaling pathway. The layered diagram further illustrated the detailed cellular communication network within the TGFb signaling pathway. The C4 *TTN+* plasma cell subtype primarily received its own signals through an autocrine pathway and established paracrine connections with megakaryocytes and HSCs (**Figure 5D**). In this signaling pathway, C4 *TTN+* plasma cell subtype served primarily as a signal receiver, while megakaryocytes functioned as the main signal sender, with megakaryocytes, myeloid cells, and MEPs acting as influencers participating in the signal communication process (**Figure 5E**). Subsequently, the violin plots were used to further compare the expression of ligand-receptor pairs in the TGFb signaling pathway (**Figure 5F**). We found that the C4 *TTN+* plasma cell subtype, megakaryocytes, and HSCs exhibited close interactions within the TGFB1-(ACVR1C+TGFBR2) ligand-receptor communication network (**Figure 5G**).

### 3.5 Inference of TF regulatory networks using SCENIC

**Figure 6:**
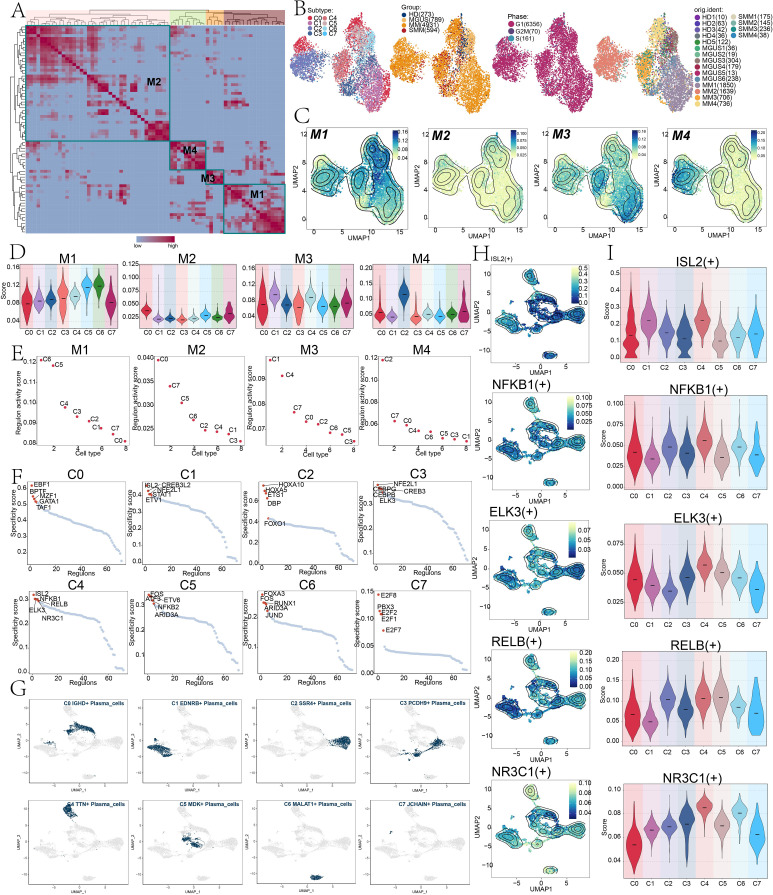
Construction of TF regulatory network and identification of key regulatory modules. **(A)** Heatmap using the SCENIC algorithm and AUCell scores to classify plasma cell subtypes regulatory factors into four modules. **(B)** The UMAP plots were colored myeloma-associated plasma cells according to their regulatory module scores. The distribution of plasma cell subtypes, groups, phases, and sample sources were depicted from left to right. **(C)** Contour plots showed the expression levels and scores of TFs in the four regulatory modules. **(D)** Violin plots compared the AUCell scores of different plasma cell subtypes in M1, M2, M3, and M4. **(E)** Scatter plots showed the RAS score rankings of TFs in the four modules for eight plasma cell subtypes. **(F)** Scatter plots showed the specificity score rankings of the top five TFs in the eight plasma cell subtypes. **(G)** The UMAP plots showed the distribution of the plasma cell subtypes. **(H)** Contour plots depicted the distribution of ISL2(+), NFKB1(+), ELK3(+), RELB(+), and NR3C1(+) in UMAP plots. **(I)** Violin plots compared the expression levels of ISL2(+), NFKB1(+), ELK3(+), RELB(+), and NR3C1(+) in eight plasma cell subtypes.

To systematically analyze the transcriptional regulatory features of plasma cell subtypes, we employed the SCENIC tool, integrated with the AUCell algorithm, to group key TFs into four regulatory modules. TFs within each module exhibited similar functional properties and expression patterns (**Figure 6A**). We then colored the UMAP plots, clustered by activity scores based on regulatory modules, by subtypes, groups, phases, and sample sources, as shown in **Figure 6B**. **Figures 6C–E** showed the expression distribution of TFs within each regulatory module and compared the AUCell scores and regulatory activity scores across different plasma cell subtypes within each module. The results showed that C4 *TTN+* plasma cell subtype exhibited relatively higher expression levels in the M3 module, indicating that C4 *TTN+* plasma cell subtype was primarily regulated by the TFs present in M3. We then identified the top five TFs for eight plasma cell subtypes and presented their distribution in UMAP plots (**Figures 6F, G**).

We identified the top five TFs in C4 *TTN+* plasma cell subtype as ISL2, NFKB1, ELK3, RELB, and NR3C1. We next visualized the expression of ISL2 (+), NFKB1 (+), ELK3 (+), RELB (+), and NR3C1 (+) in contour plots (**Figure 6H**) and compared their AUCell scores in each plasma cell subtype using violin plots (**Figure 6I**). Notably, *ELK3* had a relatively high specificity score in C4 *TTN+* plasma cell subtype and displayed elevated expression levels in M3. Based on these findings, we inferred that the ELK3 may be key to the C4 *TTN+* plasma cell subtype transcriptional regulatory network. To test this hypothesis, we then conducted *in vitro* experiments.

### 3.6 The *ELK3* knockdown suppressed proliferation, colony formation, migration, and survival of MM plasma cells

**Figure 7:**
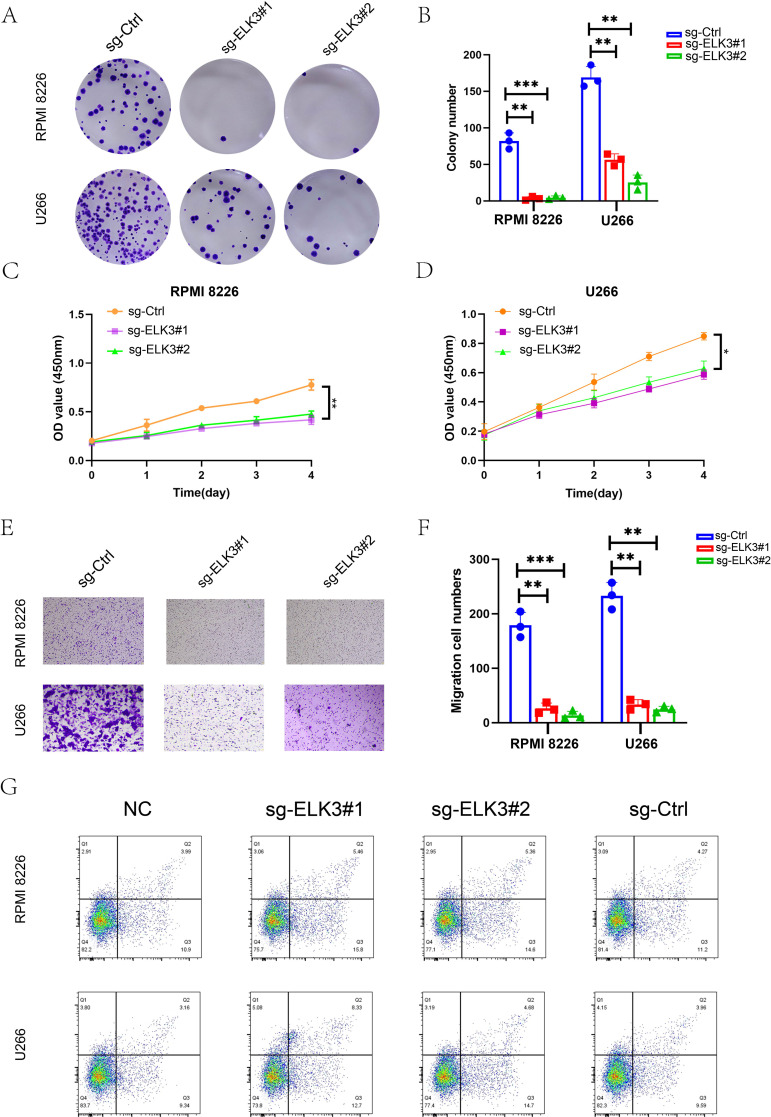
*In vitro* experiments, verification of *ELK3* knockdown efficiency and quantitative analysis of cell apoptosis. **(A)** Representative images of colony formation assays in RPMI 8226 and U266 cells transfected with sg-Ctrl, sg-ELK3#1, or sg-ELK3#2. **(B)** Quantification of colony numbers, quantitative analysis revealed that colonies formed by sg-ELK3#1 and sg-ELK3#2 groups were significantly fewer than those of the control (sg-Ctrl) group (p<0.01). **(C, D)** CCK-8 assays showing cell proliferation curves of RPMI 8226 **(C)** and U266 **(D)** cells over four days. The OD_450_ values reflected cell viability. **(E)** Representative images of Transwell migration assays for indicated groups. **(F)** Quantification of migrated cell numbers per field demonstrated a significant reduction in the sg-ELK3 groups (p<0.01). **(G)** Flow cytometry analysis of apoptosis using Annexin V–FITC/PI staining in RPMI 8226 and U266 cells. Cells were grouped as NC, sg-Ctrl, sg-ELK3#1 and sg-ELK3#2. Data were presented as mean ± SD (n = 3). Statistical significance was determined by one-way ANOVA with Tukey’s *post-hoc* test; p < 0.05(*), p < 0.01 (**), and p < 0.001 (***).

To validate the function of *ELK3* in proliferating malignant plasma cell subtypes, we next evaluated the *ELK3* knockdown in RPMI 8226 and U266 cell lines. **Figure 7A** showed the results of the colony formation assays, indicating that the *ELK3* silencing significantly reduced clonogenic capacity in both RPMI 8226 and U266 cell lines. Furthermore, quantitative analysis demonstrated that the number of colonies formed by the sg-ELK3#1 and sg-ELK3#2 groups was markedly reduced compared with that of the sg-Ctrl group (**Figure 7B**), indicating a significant impairment in long-term proliferative capacity. Similarly, CCK-8 proliferation assays showed that *ELK3* depletion substantially decreased cell viability in a time-dependent manner (**Figures 7C, D**). The OD_450_ values were consistently lower in the sg-ELK3 groups than in the sg-Ctrl groups, indicating that *ELK3* promoted the growth of MM plasma cells. Transwell migration assays further revealed that loss of *ELK3* markedly suppressed the motility of MM plasma cells (**Figure 7E**). Quantification of migrated cells demonstrated a significant reduction in the *sg-ELK3* groups (**Figure 7F**), suggesting that *ELK3* contributed to the invasive phenotype of MM.

Flow cytometric analysis using Annexin V–FITC/PI staining was performed to evaluate apoptosis following *ELK3* depletion. As shown in **Figure 7G**, sg-ELK3#1 and sg-ELK3#2 markedly increased the proportion of apoptotic cells in both RPMI-8226 and U266 cell lines compared with the sg-Ctrl group. In addition, the NC group exhibited a similar apoptotic profile to that of the sg-Ctrl group, indicating that the sgRNA delivery system itself did not significantly affect basal cell viability. These results demonstrated that the elevated apoptosis is specifically attributable to *ELK3* depletion rather than to nonspecific effects of the vector or transduction procedure.

Collectively, these findings confirmed that *ELK3* was highly expressed in proliferating malignant plasma cell clusters, further validating the conclusions in scRNA-seq. *ELK3* promoted the proliferation, migration, and survival of MM plasma cells and was associated with the progression of MM.

## 4 Discussion

The occurrence of MM is associated with multiple factors, including age, gender, and family history (55), but its exact etiology remains unclear. With the continuous optimization of diagnostic and treatment strategies, progression-free and overall survival have been significantly improved in MM patients (56). However, MM remains difficult to cure (57). The emergence and evolution of refractory clones, as well as complications and treatment-related toxicities, further exacerbate the poor prognosis of MM patients (58). Notably, MGUS and SMM, as premalignant stages of MM, exhibit significant initial genetic heterogeneity (59). This heterogeneity suggests that it is urgent and necessary to further explore the biological characteristics of MM and its precancerous stages and elucidate the molecular mechanisms regulating MM clonal diversity (60).

In this study, we systematically analyzed bone marrow aspiration samples from SMM, MM, HD, and MGUS using scRNA-seq technology. We systematically characterized the cellular heterogeneity during MM progression at the single-cell level, and constructed a high-resolution cellular atlas. A total of 10 distinct cell types were identified. Notably, plasma cells were significantly enriched in MM samples, reflecting the characteristic proliferation of malignant plasma cells, a hallmark of the disease state (61).

An increasing body of evidence indicates that malignant plasma cells in MM exhibit pronounced transcriptional heterogeneity, which is closely associated with disease progression and therapeutic resistance (62–64). Consistent with previous studies, we concentrated more on the heterogeneity of plasma cells. We first identified a tumor-associated plasma cell subtype—the C4 *TTN+* plasma cell subtype. The top 5 marker genes for this subtype were *IGHV4-59, FRZB, IGHG1, IGHG3*, and *IGHG4*. In addition, as *TTN* was expressed at a relatively higher level in this subtype, it was selected as the named gene for its designation.

*TTN* gene contains 364 exons (65). Owing to its exceptionally large size, *TTN* is prone to mutations and is recognized as a key pathogenic gene in several cardiovascular and musculoskeletal disorders (66–68). Moreover, pan-cancer analyses have identified *TTN* as one of the most frequently mutated genes across multiple tumor types (69). Previous studies have suggested that expression heterogeneity associated with *TTN* mutations may correlate with prolonged survival in patients with lung adenocarcinoma (70, 71) and may enhance the sensitivity of rectal adenocarcinoma to radiotherapy (72). Conversely, other studies have reported that the expression of *TTN* and its related long non-coding RNA (*lncRNA-TTN-AS1*) may promote the development and metastasis of melanoma (73). In MM, *TTN* has also been identified as a frequently mutated gene, and its mutations have been associated with unfavorable prognosis (74, 75). Collectively, these findings indicate that *TTN* is closely associated with tumor mutational burden and the progression of multiple malignancies (76), providing supporting evidence for the potential biological relevance of the higher *TTN* expression observed in this study.

At the sample level, the C4 *TTN+* plasma cell subtype was present in both SMM and MM, further supporting its identification as a tumor-associated plasma cell subtype. Enrichment analysis results demonstrated that the C4 *TTN+* plasma cell subtype was primarily involved in biological processes such as metallopeptidase activity, enzyme inhibitor activity, mRNA 5’-UTR binding, and structural constituents of the ribosome. Next, we presented the top 20 metabolic pathways associated with the C4 *TTN+* plasma cell subtype, among which oxidative phosphorylation, N-glycan biosynthesis, and glutathione metabolism played key roles in its metabolic network. The flexible metabolic profile of the C4 *TTN+* plasma cell subtype offered considerable adaptation to changes in the TME in addition to providing enough energy for fast tumor cell proliferation. Additionally, this metabolic flexibility was strongly linked to relapse following MM treatment and may contribute to the emergence of drug resistance (77).

CytoTRACE2 analysis was used to predict the differentiation potential of plasma cells. Results showed that the C4 *TTN+* plasma cell subtype exhibited a relatively high cytoTRACE2 score, indicating relatively strong differentiation potential but a low degree of differentiation, potentially representing an activated malignant plasma cell population. Subsequently, we further inferred the pseudotime trajectory of MM through Monocle and Slingshot analyses. The C4 *TTN+* plasma cell subtype was primarily distributed at the end of Lineage 4. Based on the sample origin and metabolic characteristics of the C4 *TTN+* plasma cell subtype, we suggested that lineage 4 may represent a differentiation trajectory associated with tumor progression, and that the C4 *TTN+* plasma cell subtype may be associated with the malignant characteristics of MM.

The expression of stemness-related genes can affect the occurrence, development, metastasis, and recurrence of cancer (78, 79). In this study, we observed elevated expression of multiple stemness-related genes in C4 *TTN+* plasma cell subtype, particularly *CD44, HIF1A*, *ABCG2, KDM5B*, and *BMI1*. This suggested that this subtype may possess strong stemness characteristics. Studies have shown that *CD44* drives the transduction of multiple cancer-related signals and is widely considered a cancer stem cell marker in various cancers (80). *HIF1A* expression under hypoxic conditions activates multiple oncogenes, promoting cancer progression through multiple mechanisms and being associated with higher mortality (81, 82). *ABCG2* expression levels are significantly correlated with tumor stemness scores and exhibit multifunctionality in various cancers. It is believed to have a protective effect in some cancers (83) and may be associated with multidrug resistance in cancers (84, 85). *KDM5B* may regulate the expression of tumor suppressor genes and oncogenes to some extent, thereby affecting the proliferation and differentiation of cancer cells (86, 87). *BMI1* is overexpressed in a number of malignancies and linked to treatment resistance, which results in a worse prognosis and a greater cancer grade or stage (88, 89). In summary, the elevated expression of these key stemness-related genes in the C4 *TTN+* plasma cell subtype suggested that this subtype may possess strong stemness and invasiveness characteristics. This finding further supported the speculation that the C4 *TTN+* plasma cell subtype was a tumor-associated plasma subtype.

CellChat analysis suggested that a complex intercellular communication network existed between the C4 *TTN+* plasma cell subtype and multiple cell types in the MM TME. The TGFb signaling pathway may play a pivotal role in the cellular interaction network of the C4 *TTN+* plasma cell subtype. Further ligand-receptor analysis revealed that TGFB1-(ACVR1C+TGFBR2) made a significant contribution to mediating communication between C4 *TTN+* plasma cell subtype and megakaryocytes, thereby supplementing and refining the cell interaction map in MM-TME. Previous studies have shown that the TGFb signaling pathway affects the development and function of immune cells (90), and promotes the progression of malignant tumors through multiple mechanisms such as inducing immunosuppression, epithelial-mesenchymal transition, and promoting lymphangiogenesis (91). In addition, megakaryocytes can not only support the growth of malignant plasma cells in the benign bone marrow microenvironment (92), but also dynamically regulate the functional state of HSCs and promote the regeneration of HSCs after injury (93). It is worth noting that an increase in myeloid-biased HSCs (my-HSCs) in HSCs has been shown to weaken adaptive immune responses (94). In summary, during the progression of MM, the C4 *TTN+* plasma cell subtype may influence the functional states of megakaryocytes and HSCs through the TGFb signaling pathway, and may further be linked to adaptive immune dysregulation and inflammatory responses. However, the precise regulatory mechanisms underlying these associations remain unclear and require further experimental validation.

Our study further constructed a high-resolution map of the TF regulatory network in MM. TFs are often persistently activated at multiple stages of cancer progression, promoting tumor growth and invasion (95). With the continuous advancement of molecular biology research, an increasing number of TFs have been identified as cancer drivers (96), and their aberrant expression or activation is considered a key factor in the development of various malignancies. Therefore, identifying and targeting key TF is crucial for curbing the progression of MM.

In this study, we categorized the transcriptional regulation patterns of eight plasma cell subtypes into four regulatory modules using the SCENIC method and AUCell scores. The results showed that the C4 *TTN+* plasma cell subtype was primarily regulated by the M3 module. Based on the specificity score, we identified the top five key TFs for the C4 *TTN+* plasma cell subtype: ISL2, NFKB1, ELK3, RELB, and NR3C1. Studies have shown that ELK3 is associated with the occurrence and development of various cancers and can promote the invasion and metastasis of cancer cells, playing a carcinogenic role in glioma (97, 98), triple-negative breast cancer (99), gastric cancer (100), and pancreatic cancer (101). Given the relatively high expression of ELK3 in the C4 *TTN+* plasma cell subtype, we postulated that it might have a similar function in the progression of MM. To verify this hypothesis, we conducted further *in vitro* experiments.

Subsequently, we further confirmed through *in vitro* experiments that *ELK3* can promote the growth, migration, and survival of MM plasma cells. We found that *ELK3* silencing in RPMI 8226 and U266 cells decreased colony number, proliferation rate, and cell viability as well as inhibited plasma cell invasion and migration, according to assays for colony formation, CCK-8 proliferation, and Transwell migration assays. Flow cytometry results further supported the role of *ELK3* in promoting MM plasma cell survival. *ELK3* may be associated with enhanced proliferative and invasive phenotypes of plasma cells, indicating its potential as a therapeutic target in MM and offering new possibilities for targeted intervention.

This study revealed the dynamic changes at the molecular level during the progression of MM and proposed a novel target, ELK3, with potential clinical applications. This offered a new possibility for targeted therapy of MM. However, there are still several limitations. First, the scRNA-seq data in this study were derived exclusively from the GSE271107, which included a limited number of samples. This may have introduced potential sampling bias and could affect the generalizability of the findings. Second, the analysis was based only on single-cell sequencing and transcriptomic data and was not validated using multi-omics methods. Third, the functional validation experiments were conducted *in vitro* and may not fully recapitulate the complex bone marrow microenvironment *in vivo*. Consequently, future studies incorporating larger multicenter cohorts and multi-omics approaches are warranted to further validate and extend these findings.

## 5 Conclusion

This study employed scRNA-seq to characterize the cellular heterogeneity associated with the progression of MM. We identified a tumor-associated plasma cell subpopulation, the C4 *TTN+* plasma cell subtype. This subtype may potentially interact with megakaryocytes and HSCs through the TGFb signaling pathway and may be associated with adaptive immune dysregulation and inflammatory responses in MM. Further analyses suggested that the TF ELK3 may be involved in the progression of MM. These findings contribute to a deeper understanding of the molecular features underlying MM progression and may provide new perspectives for the development of targeted therapeutic strategies.

## Data Availability

The original contributions presented in the study are included in the article/supplementary material. Further inquiries can be directed to the corresponding authors.
